# Synthesis, Reactions and Evaluation of the Antimicrobial Activity of Some 4-(*p*-Halophenyl)-4*H*-naphthopyran, Pyranopyrimidine and Pyranotriazolopyrimidine Derivatives

**DOI:** 10.3390/ph5070745

**Published:** 2012-07-12

**Authors:** Ashraf H. F. Abd El-Wahab

**Affiliations:** Chemistry Department, Faculty of Science, Al-Azhar University, Nasr City, Cairo 11884, Egypt; Email: ash_abdelwahab@hotmail.com; ash_abdelwahab@yahoo.com; Tel: +20-10-0819-9893; Fax: +20-22-262-958

**Keywords:** antimicrobial activity, arylidienemalonitrile, 6-methoxy-2-naphthol, naphthopyranopyrimidine, naphthopyranotriazolopyrimidine, carboxylic acid derivatives

## Abstract

A series of naphthopyran derivatives **3a–f** were prepared. Reaction of *2-*amino-4-(*p*-chlorophenyl)-7-methoxy-4*H*-naphtho[2,1-*b*]pyran-3-carbonitrile (**3b**) with Ac_2_O afforded two products, 2-acetylamino-7-methoxy-4-(*p*-chlorophenyl)-4*H*-naphtho-[2,1-*b*]pyran-3-carbonitrile (**4**) and 10,11-dihydro-3-methoxy-9-methyl-12-(*p*-chloro-phenyl)-12*H*-naphtho[2,1-*b*]pyran[2,3-*d*]pyrimidine-11-one (**5**) and treatment of **3b** with benzoyl chloride gave the pyranopyrimidin-11-one derivative **6**. While treatment of **3b** with formamide afforded 11-amino-3-methoxy-12-(*p*-chlorophenyl)-12*H*-naphtho[2,1-*b*]pyrano[2,3-*d*]pyrimidine (**7**). Reaction of **3b** with triethyl orthoformate gave the corresponding 2-ethoxymethyleneamino-7-methoxy-4-(*p*-chlorophenyl)-4*H*-naphtho-[2,1-*b*]pyran-3-carbonitrile (**8**). Hydrazinolysis of **8** in EtOH at room temperature yielded 10-amino-10,11-dihydro-11-imino-3-methoxy-12-(*p*-chlorophenyl)-12*H*-naphtho[2,1-b]pyrano-[2,3-d]pyrimidine (**9**), while aminolysis of **8** with methylamine or dimethylamine gave the corresponding pyranopyrimidine and *N,N*-dimethylaminomethylene derivatives **10** and **11**. Condensation of **9** with some carboxylic acid derivatives afforded triazolopyrimidine derivatives **12–16**, while reaction of **9** with benzaldehyde gave 10-benzalamino-10,11-dihydro-11-imino-3-methoxy-12-(*p*-chlorophenyl)12*H*-naphtho[2,1-*b*]pyrano[2,3-*d*]pyrimidine (**17**). The structures of the newly synthesized compounds were confirmed by spectral data. The synthesized compounds were also screened for their antimicrobial activity.

## 1. Introduction

Pyran and fused 4*H*-pyran derivatives have attracted a great deal of interest owing to their antimicrobial activity [1,2,3,4,5], inhibition of influenza, virus sialidases [6], mutagenic activity [7], activity as antiviral [8] and antiproliferation agents [9], sex-pheromones [10], antitumor [11] and anti-inflammatory agents [12]. Moreover, pyran derivatives are well known for their antihistaminic activity [13]. Also pyrimdines are an important class of compounds and have widespread applications from pharmaceuticals to materials [14], with activities such as Tie-2 kinase inhibitors [15], HIV-1 inhibitor [16], anti-malarial [17], adenosine A1 receptor antagonism [18], anticancer [19], analgesic [20], cardiovascular [21] and antiallergic activities [22]. In view of the important biological properties of the pyran and pyrimidine derivatives as medicinal agents, we report here the synthesis and antimicrobial activities of new naphthopyrano, naphthopyranopyrimidine and naphthopyranotriazolopyrimidine derivatives.

## 2. Results and Discussion

**Scheme 1:**
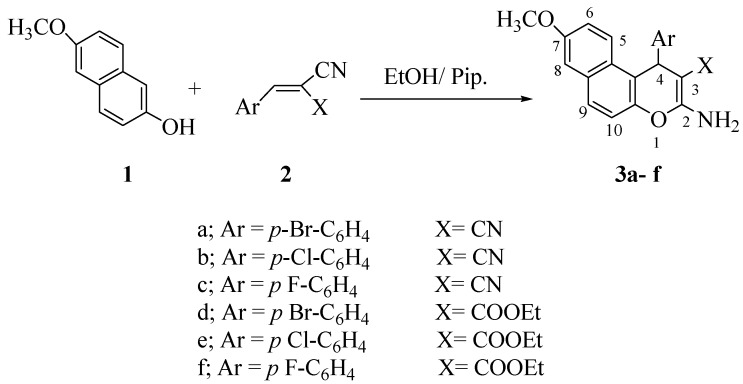
Synthesis of naphthopyran derivatives **3a–f**.

Condensation of 6-methoxy-2-naphthol (**1**) with substituted 4-halobenzylidenmalononitriles **2a–c** and/or ethyl 4-halobenzylidenmalonates **2d–f** afforded the corresponding 2-amino-4-(*p*-halophenyl)-7-methoxy-4*H*-naphtho[2,1-*b*]pyran-3-carbonitriles **3a–c** and ethyl-2-amino-4-(*p*-halophenyl)-7-methoxy-4*H*-naphtho[2,1-*b*]pyran-3-carboxylates **3d–f**, respectively [23,24] (Scheme 1).

The structure of compounds **3a–f** were established by spectral data. The IR spectrum of compounds **3a–f** showed absorptions at 3,466–3,350, 3,346–3,314 cm^−1^ (NH_2_), 3,192–3,000 cm^−1^ (CH-aromatic), 2,950–2,900 (CH-aliphatic), 2,200–2,192 cm^−1^ (C≡N), 1,682–1,670 cm^−1^ (C=O). The ^1^H-NMR of compounds **3a–f** showed chemical shifts δ_H_ at 3.75–3.78 (s, 3H, OCH_3_), 5.27–5.530 (s, 1H, pyran CH), 6.98–7.21 (br, 2H, NH_2_, exchangeable by D_2_O) while the ^13^C-NMR of compound **3b** showed δ_C_ at 56.5 (OCH_3_), 28.4 (C-4), 117.1 (C≡N) and compound **3e** showed chemical shifts δ_C_ at 13.1 (CH_3_-ester), 28.4 (C-4), 56.5 (OCH_3_), 62.5 (CH_2_-ester) and 172.2 (CO).

**Scheme 2:**
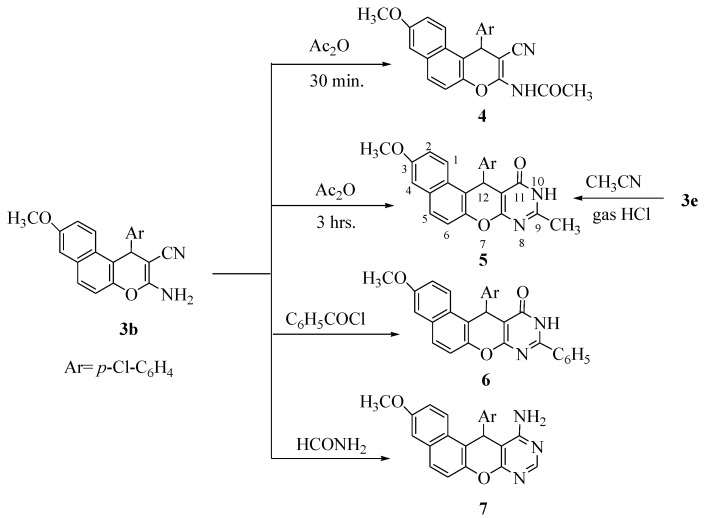
Synthesis of naphthopyran and naphthopyranpyrimidine derivatives **4–7**.

Treatment of **3b** with Ac_2_O gave two products, depending on the reaction time; one product was identified as 2-acetylamino-7-methoxy-4-(*p*-chlorophenyl)-4*H*-naphtho[2,1-*b*]pyran-3-carbonitrile (**4**, 1/2 hour), while the other was identified as 10,11-dihydro-3-methoxy-9-methyl-12-(*p*-chlorophenyl)-12*H*-naphtho[2,1-*b*]pyrano-[2,3-*d*]pyrimidine-11-one (**5**, 3 hours). Support for structure **5** was obtained by its independent synthesis by the reaction of **3e** with CH_3_CN in the presence of dry HCl gas [25]. Reaction of **3b** with benzoyl chloride gave the pyranopyrimidin-11-one derivative **6**, while with formamide afforded 11-amino-3-methoxy-12-(*p*-chlorophenyl)-12*H*-naphtho[2,1-*b*]pyrano[2,3-d]pyrimidine **7** (Scheme 2).

The structure of compounds **4**–**7** were established from their spectral data. The IR spectrum of compound **4** showed absorptions at 3,400 (NH), 2,202 cm^−1^ (CN), 1,612 cm^−1^ (C=O), while compound **5** showed absorptions at 3,464 (NH), 1,650 cm^−1^ (C=O) and compound **7** showed υ at 3,458, 3,380 (NH_2_). The ^1^H-NMR of compounds **4–7** showed chemical shifts δ_H_ at 3.78–3.82 (s, 3H, OCH_3_), 5.66–5.70 (s, 1H, pyran CH). The mass spectra of compounds **6** and **7** provided additional evidence for the proposed structures.

**Scheme 3:**
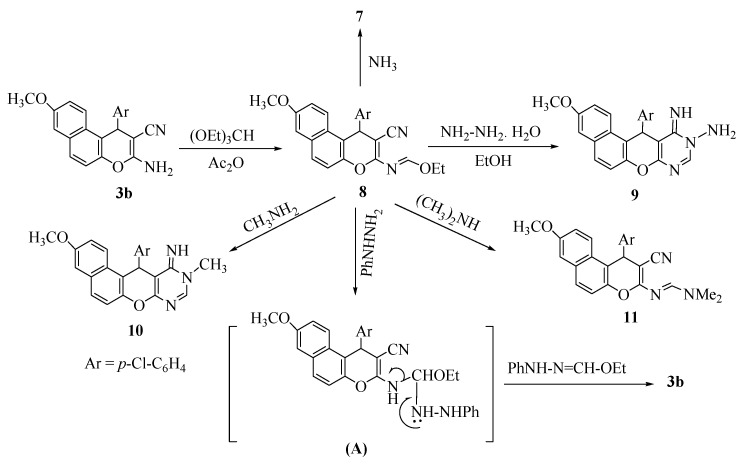
Reaction of **3b** with triethyl orthoformate and of ammionium derivatives.

Reaction of **3b** with triethyl orthoformate gave the corresponding 2-ethoxymetheneamino derivative **8**. Hydrazinolysis of **8** in EtOH at room temperature yielded 10-amino-10,11-dihydro-11-imino-3-methoxy-12-(*p-*chlorophenyl)-12*H*-naphtho[2,1-*b*]pyrano[2,3-*d*]pyrimidine (**9**). During treatment of **8** with phenylhydrazine, an nonisolable addition product **A** was formed first, followed by elimination of the nonisolable ethyl formatephenylhydrazone to give the enaminonitrile **3b**. Ammonolysis of **8** in MeOH at room temperature afforded compound **7** and aminolysis of **8** with methylamine and/or dimethylamine gave the corresponding 10-methyl-pyranopyrimidine and *N,N*-dimethylamino-methylene derivatives **10** and **11**, respectively (Scheme 3).

The structure of compounds **8–11** were established from their spectral data. The IR spectrum of compound **8** showed absorptions at 2,203 cm^−1^ (C≡N), while compound **9** showed υ at 3,316, 3,270 (NH_2_), 3,209 cm^−1^ (NH) and compound **11** showed an absorption at 2,204 cm^−1^ (C≡N). The ^1^H-NMR of compounds **8–11** showed chemical shifts δ_H_ at 3.77–3.80 (s, 3H, OCH_3_) and 5.29–5.58 (s, 1H, pyran CH).

**Scheme 4:**
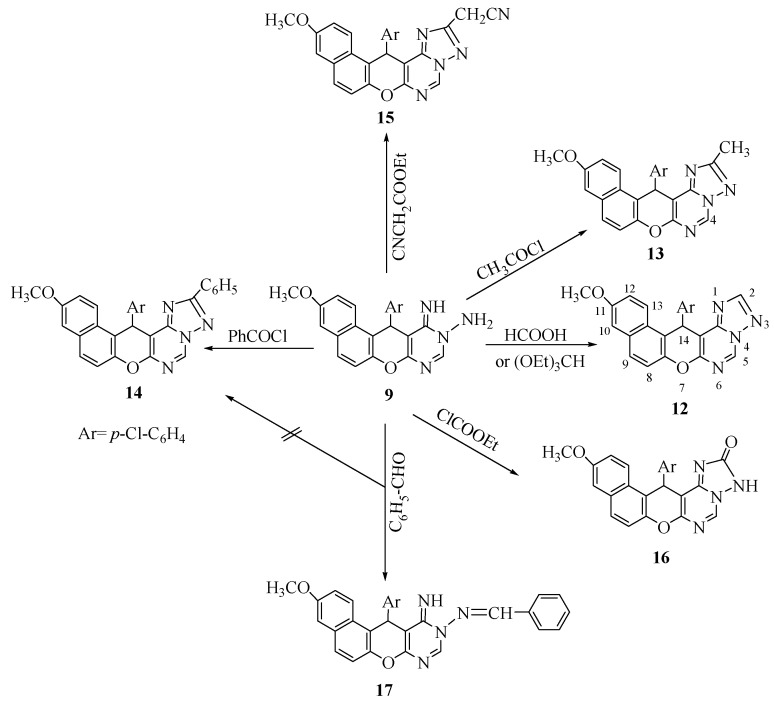
Synthesis of naphthopyrantriazolopyridimide derivatives **12–17**.

Reaction of **9** with formic acid or triethyl orthoformate, acetylchloride and benzoyl chloride afforded the corresponding triazolopyrimidine derivatives **12–14**, while cyclocondensation of **9** with ethyl cyanoacetate gave the corresponding 2-cyanomethyl derivative **15**. Treatment of **9** with ethyl chloroformate in dry benzene affored traizolo-2-one derivative **16**. Reaction of **9** with benzaldehyde gave 10-benzalamino-10,11-dihydro-11-imino-3-methoxy-12-(*p*-chlorophenyl)-12*H*-naphtho-[2,1-*b*]-pyrano[2,3-*d*]pyrimidine (**17**) instead of the expected triazolopyrimidine derivative **14** (Scheme 4).

The structure of compounds **12–17** were established from their spectral data. The IR spectrum of compound **15** showed an absorption at 2,200 cm^−1^ (C≡N), compound **16** showed absorptions at 3,200 cm^−1^ (NH) and 1,638 cm^−1^ (C=O), while compound **17** showed absorptions at 3,261 cm^−1^ (NH), and 1,621 cm^−1^ (C=N). The ^1^H-NMR of compounds **12–17** showed chemical shifts δ_H_ at 3.78–3.80 (s, 3H, OCH_3_), 526–5.66 (s, 1H, pyran CH), 8.63–9.64 (s, 1H, pyrimidine CH).

The antimicrobial activity of the newly synthesized compounds **3–17** was evaluated against the bacterial strains *Staphylococcus aureus (NCTC-7447)*, *Bacillus cereus (ATCC-14579)*, *Escherichia coli (NCTC-10410)*, *Serratia marcescens (IMRU-70) *and the fungal strains *Aspergillus fumigatus (MTCC-3008)*, and *Candida albicans* (MTCC-227) by the disk diffusion method [26,27]. Ampicillin and ketoconazole were used as standard drugs for the bacteria and fungi, respectively. Preliminary screening of the naphthopyran derivatives and standard drugs was performed at fixed concentrations of 500 μg/mL. Inhibition was recorded by measuring the diameter of the inhibition zone at the end of 24 hours for bacteria and 72 hours for fungi. Each experiment was repeated twice. Based on the results of zone of inhibition, the minimum inhibitory concentration (MIC) of compounds **3–17** against all bacterial and fungal strains was determined by the liquid dilution method. Stock solutions of the tested compounds with 500, 250, 200, 100, 50, 25, 12.5, and 6.25 μg/mL concentrations were prepared with DMSO as solvent. The solutions of standard drugs, ampicillin and ketoconazole, were prepared in the same concentrations. The minimum inhibitory concentration at which no growth was observed was taken as the MIC value (Table 1). The comparison of the MICs (μg/mL) of potent compounds and standard drugs against tested strains are presented in Table 1. Investigation of the antibacterial screening data showed that some of the compounds were active against all four pathogenic bacteria. Ethyl 2-amino-4-(*p*-chlorophenyl)naphthopyrane-3-carboxylate (**3e**), triazolopyrimidine derivative **12**, 2-methyl-triazolopyrimidine derivative **13**, 2-phenyl-triazolopyrimidine derivative **14** and, 2-oxa-triazolopyrimidine derivative **16** exhibited good activity against *S. aureus*. Similarly 2-amino-3-cyano-4-(*p*-flourophenyl)naphthopyran (**3c**), 2-methyltriazolopyrimidine derivative **14**, triazolopyrimidine derivative **12**, triazolopyrimidine 2-ethanenitrile derivative **15** and 2-oxa-triazolopyrimidine derivative **16** exhibited good activity against *B. cereus* and 2-amino-3-cyano4-(*p*-bromophenyl)naphthopyran (**3a**), 2-amino-3-cyano-4-(*p*-chlorophenyl)naphthopyran (**3b**), 2-amino-3-cyano-4-(*p*-flourophenyl)naphthopyran **3c**, ethyl 2-amino-4-(*p*-chlorophenyl)naphthopyran-3-carboxylate (**3e**), 2-methyl-triazolopyrimidine derivative **13** and triazolopyrimidine 2-ethanenitrile derivative **16** exhibited good activity against *E. coli*, while 2-amino-3-cyano-4-(*p*-bromo/chloro/fluorophenyl)naphthopyran derivatives **3a–c**, triazolopyrimidine derivative **12**, 2-phenyltriazolopyrimidine derivative **14** and 2-oxatriazolopyrimidine derivative **16** exhibited good activity against *S. marcescens*. Aminoimino derivative **9** was inactive against *S. aureus*, while the pyranpyrimidin-11-one **6** and 10-benzalamino-pyranopyrimidine derivative **17** was inactive against *B. cereus*, the compounds ethyl 2-amino-4-(*p*-chlorophenyl)naphthopyrane-3-carboxylate (**3e**), 11-amino-pyranopyrimidine **7** and 2-ethoxy-methyleneamino derivative **8** was inactive against *E. coli*, and the 2-acetylamino-pyranopyrimidine compound **4** was inactive against *S. marcescens*. The remaining compounds showed moderate to weak antibacterial activity.

**Table 1:** Antimicrobial activity of the new compounds*.*

Compounds	Minimum inhibitory concentration (MIC) in μg/mL					
	Bacterial strains				Fungal strains	
	*S. aureus*	*B. cereus*	*E. coli*	*S. marcescens*	*A. fumigatus*	*C. albicans*
**3a**	100	100	25	50	100	100
**3b**	125	200	50	50	125	100
**3c**	100	50	25	50	50	50
**3d**	250	200	500	500	250	100
**3e**	25	100	50	100	25	50
**3f**	500	500	-	125	250	500
**4**	500	250	250	100	500	-
**5**	100	125	-	500	-	250
**6**	100	-	100	250	500	500
**7**	500	200	-	200	-	250
**8**	250	200	500	500	250	100
**9**	-	500	-	100	500	500
**10**	500	250	100	250	250	500
**11**	-	500	250	-	-	500
**12**	25	25	50	25	100	50
**13**	25	50	25	50	50	500
**14**	50	100	100	25	25	50
**15**	100	25	25	125	100	500
**16**	50	25	50	50	125	100
**17**	500	-	250	500	-	-
**Ampcillin**	6.25	6.25	6.25	6.25	6.25	6.25
**Ketoconazole**	-	-	-	-	31.25	31.25

The antifungal results (Table 1) revealed that the synthesized compounds showed variable degrees of inhibition against the tested fungi. The compounds 2-amino-3-cyano-4-(*p*-fluorophenyl)naphthopyran (**3c**), ethyl 2-amino-4-(*p*-chlorophenyl)naphthopyran-3-carboxylate (**3e**), and 2-phenyltriazolopyrimidine **14** possessed good antifungal activity against *A. fumigatus *and *C. albicans*, while the compounds 9-methyl-pyranopyrimidine **5**, 11-amino-pyranopyrimidine **7**, *N*,*N*-dimethylaminomethylene derivative **11** and 10-benzalaminopyranopyrimidine **17** were inactive against *A. fumigates*, and the 2-acetylaminopyranopyrimidine **4** and 10-benzalaminopyranopyrimidine **17** were inactive against *C. albicans.* The remaining compounds showed moderate to weak antifungal activity.

## 3. Experimental

### 3.1. General

Melting points were determined on a Stuart melting point apparatus and are uncorrected. IR spectra (ν, cm^−1^) were recorded in KBr using a FT-IR 5300 spectrometer and aPerkin Elmer spectrum RXIFT-IR system. The ^1^H-NMR at (300 MHz) and ^13^C-NMR spectra (75 MHz) were recorded in DMSO-d_6_ on a Varian Mercury VX-300 NMR spectrometer. Chemical shifts (δ) are referred to that of the solvent. Mass spectra were measured on a Shimadzu GMMS-QP-1000 EX mass spectrometer at 70 eV. The elemental analyses were performed at the Micro Analytical Center, Cairo University, Egypt.

### 3.2. General Procedure: Synthesis of 4H-Pyran Derivatives ***3a–f***

A mixture of substituted 4-halobenzylidenmalononitriles **2a**–**c** (10 mmol) and/or ethyl 4-halobenzylidenmalonates **2d**–**f** (10 mmol), 6-methoxy-2-naphthol (**1**) (0.17 g, 10 mmol) and piperidine (0.5 mL) in absolute EtOH (50 mL) was heated until precipitation was completed. The precipitate was collected by filtration and recrystallized from dioxane and EtOH/benzene respectively.

*2-Amino-4-(p-bromophenyl)-7-methoxy-4H-naphtho[2,1-b]pyrane-3-carbonnitrile *(**3a**). White crystals (dioxane); yield 88%, mp 260–262 °C; IR: 3,454, 3,315 (NH_2_), 3,182 (CH-aromatic), 2,950 (CH-aliphatic), 2,192 (C≡N), 1,658 (C=C). ^1^H-NMR δ: 3.78 (s, 3H, OCH_3_), 5.27 (s, 1H, pyran CH), 6.98 (br, 2H, NH_2_, exchangeable by D_2_O), 7.06–7.79 (m, 9H, Ar-H). Anal. Calcd. for C_21_H_15_BrN_2_O_2_ (406.03): C, 61.90; H, 3.90; N, 6.84%. Found: C, 61.93; H, 3.71; N, 6.88%.

*2-Amino-4-(p-chlorophenyl)-7-methoxy-4H-naphtho[2,1-b]pyrane-3-carbonnitrile *(**3b**). White crystals (dioxane); yield 90%, mp 246–248 °C; IR: 3,358, 3,314 (NH*_2_*), 3,186 (CH-aromatic), 2,932 (CH-aliphatic), 2,198 (C≡N), 1,660 (C=C). ^1^H-NMR δ: 3.77 (s, 3H, OCH_3_), 5.28 (s, 1H, pyran CH), 6.99 (br, 2H, NH_2_, exchangeable by D_2_O), 7.06–7.79 (m, 9H, Ar-H). ^13^C-NMR δ: 28.4 (C-4), 56.50 (OCH_3_), 59.1 (C-3), 105.9 (C-7), 117.1 (C≡N), 118.6 (C-5), 118.9 (C-9), 121.3 (C-4a), 123.6 (C-10), 127.1 (C-6), 128.2 (C-5a, C-8a), 128.7, 129.7, 131.3, 136.6, 143.8 (Ar), 150.2 (C-10), 157.2 (C-8), 160.2 (C-2). Anal. Calcd. for C_21_H_15_ClN_2_O_2_ (362.08): C, 69.52; H, 4.17; N, 7.72%. Found: C, 69.50; H, 4.15; N, 7.70%.

*2-Amino-4-(p-flouroophenyl)-7-methoxy-4H-naphtho[2,1-b]pyran-3-carbonitrile *(**3c**). White crystals (dioxane); yield 86%, mp 255–257 °C; IR: 3,466, 3,318 (NH_2_), 3,192 (CH-aromatic), 2,900 (CH-aliphatic), 2,200 (C≡N), 1,662 (C=C). ^1^H-NMR δ: 3.75 (s, 3H, OCH_3_), 5.30 (s, 1H, pyran CH), 7.01 (br, 2H, NH_2_, exchangeable by D_2_O), 7.08–8.31 (m, 9H, Ar-H). Anal. Calcd for C_21_H_15_FN_2_O_2_ (346.11): C, 72.82; H, 4.37; N, 8.09%. Found: C, 72.80; H, 4.35; N, 7.99%.

*Ethyl 2-amino-4-(p-bromophenyl)-7-methoxy-4H-naphtho[2,1-b]pyran-3-carboxylate *(**3d**). Colourless needle-like crystals (ethanol/benzene); yield 79%, mp 167–169 °C: IR: 3,350, 3,324 (NH_2_), 3,000 (CH-aromatic), 2,944 (CH-aliphatic), 1,682 (C=O), 1,618 (C=C). ^1^H-NMR δ: 1.21 (t, 3H, CH_3_, *J* = 7.1 Hz), 3.78 (s, 3H, OCH_3_), 4.04 (q, 2H, CH_2_, *J* = 7.1 Hz), 5.42 (s, 1H, pyran CH), 7.21(br, 2H, NH_2_, exchangeable by D_2_O), 7.28–7.86 (m, 9H, Ar-H). Anal. Calcd. for C_23_H_20_BrNO_4_ (453.06): C, 60.81; H, 4.44; N, 3.08%. Found: C, 60.80; H, 4.41; N, 3.03%.

*Ethyl 2-amino-4-(p-chlorophenyl)-7-methoxy-4H-naphtho[2,1-b]pyran-3-carboxylate *(**3e**). Colourless needle crystals, (ethanol/benzene); yield 82%, mp 172–174 °C: IR: 3,458, 3,324 (NH_2_), 3,010 (CH-aromatic), 2,970 (CH-aliphatic), 1,670 (C=O), 1,622 (C=C). ^1^H-NMR δ: 1.38 (t, 3H, CH_3_, *J* *=* 7.1 Hz), 3.80 (s, 3H, OCH_3_), 4.31 (q, 2H, CH_2_, *J* *=* 7.1 Hz), 5.40 (s, 1H, pyran CH), 6.24 (br, 2H, NH_2_, exchangeable by D_2_O), 6.89–7.75 (m, 9H, Ar-H). ^13^C-NMR δ: 13.1 (CH_3_-ester), 28.4 (C-4), 56.5 (OCH_3_), 59.1 (C-3), 62.5 (CH_2_-ester), 105.9 (C-7), 118.6 (C-5), 118.9 (C-9), 121.3 (C-4a), 123.6 (C-10), 127.1 (C-6), 128.2 (C-5a, C-8a), 128.7, 129.7, 131.3, 136.6, 143.8 (Ar),150.2 (C-10), 157.2 (C-8), 160.2 (C-2), 172 (C=O). Anal. Calcd. for C_23_H_20_ClNO_4_ (409.11): C, 67.40; H, 4.92; N, 3.42%. Found: C, 67.38; H, 4.90; N, 3.39%.

*Ethyl 2-amino-4-(p-flouophenyl)-7-methoxy-4H-naphtho[2,1-b]pyran-3-carboxylate *(**3f**). Colourless needle-like crystals (ethanol/benzene); yield 77%, mp 186–188 °C; IR: 3,422, 3,346 (NH_2_), 3,192 (CH-aromatic), 2,900 (CH-aliphatic), 1,682 (CO), 1,600 (C=C). ^1^H-NMR δ: 1.39 (t, 3H, CH_3_, *J* *=* 7.1 Hz), 3.78 (s, 3H, OCH_3_), 4.30 (q, 2H, CH_2_, *J* *=* 7.1 Hz), 5.54 (s, 1H, pyran CH), 6.36 (br, 2H, NH_2_, exchangeable by D_2_O), 7.05–8.06 (m, 9H, Ar-H). Anal. Calcd. for C_23_H_20_FNO_4_ (393.14): C, 70.20; H, 5.10; N, 3.52%. Found: C, 70.22; H, 5.12; N, 3.56%.

*Synthesis of 2-acetylamino-7-methoxy-4-(p-chlorophenyl)-4H-naphtho[2,1-b]pyran-3-carbonitrile *(**4**)*.* A solution of **3b** (0.36 g, 10 mmol) in Ac_2_O (20 mL) was heated under reflux for 30 min. The solid product formed was filtered off and washed with cold EtOH, The solid obtained was filtered off and recrystallized from EtOH. Pale yellow crystals, yield 89%, mp 175–177 °C; IR: 3,400 (NH), 3,122 (CH-aromatic), 2,940 (CH-aliphatic), 2,202 (C≡N), 1,612 (C=O). ^1^H-NMR δ: 2.26 (s, 3H, CH_3_), 3.82 (s, 3H, OCH_3_), 5.70 (s, 1H, pyran CH), 7.22–7.83 (m, 9H, Ar-H), 12.49 (br, 1H, NH, exchangeable by D_2_O). Anal. Calcd. for C_23_H_17_ClN_2_O_3_ (404.09): C, 68.23; H, 4.23; N, 6.92%. Found: C, 68.20; H, 4.19; N, 6.90%.

*Synthesis of 10,11-dihydro-3-methoxy-9-methyl-12-(p-chlorophenyl)-12H-naphtho-[2,1-b]pyrano[2,3-d]pyrimidine-11-one *(**5**). Method A: A solution of **3b** (0.36 g, 10 mmol) in Ac_2_O (20 mL) was heated under reflux for 3 hours. The precipitate was filtered off, washed with cold EtOH. The solid obtained was filtered off and recrystallized from DMF; Method B: Gaseous dry HCl was bubbled through the mixture of **3e** (0.40 g, 10 mmol) and CH_3_CN (30 mL) for 4–6 hours. The reaction mixture was poured into ice water and made alkaline with 10% aqueous ammonium hydroxide to give **5**. White crystals, yield 85%, mp 290–292 °C: IR: 3,464 (NH), 3,001 (CH-aromatic), 2,980 (CH-aliphatic), 1,650 (C=O), 1,620 (C=C). ^1^H-NMR δ: 2.28 (s, 3H, CH_3_), 3.78 (s, 3H, OCH_3_), 5.66 (s, 1H, pyran CH), 6.89–8.01 (m, 10H, Ar-H and NH). Anal. Calcd. for C_23_H_17_ClN_2_O_3_ (404.09): C, 68.23; H, 4.23; N, 6.92%. Found: C, 68.20; H, 4.19; N, 6.90%.

*Synthesis of 10,11-dihydro-3-methoxy-9-phenyl-12-(p-chlorophenyl)-12H-naphtho[2,1-b]-pyrano[2,3-d]pyrimidine-11-one *(**6**). A solution of **3b** (0.36 g, 10 mmol) in benzoyl chloride (20 mL) was heated under reflux for 6 hours. The excess of benzoyl chloride was removed under reduced pressure and the residue was poured into cold water. The precipitate was collected by filtration, washed with CCl_4_ (10 mL) to remove the formed benzoic acid and the residue was dried. The solid obtained was filtered off and recrystallized from DMF. Yellow crystals, yield 80%, mp > 360 °C; IR: 3,433 (NH), 3,012 (CH-aromatic), 2,892 (CH-aliphatic), 1,640 (C=O), 1,572 (C=C). MS *m/z *(%) = 466 (M+, 47.7), 326 (100), 250 (16.4), 129 (10.9). Anal. Calcd. for C_28_H_19_ClN_2_O_3_ (466.11): C, 72.03; H, 4.10; N, 6.01%. Found: C, 72.01; H, 4.02; N, 5.88%.

*Synthesis of 11-amino-3-methoxy-12-(p*-chlorophenyl*))-12H-naphtho[2,1-b]pyrano[2,3-d]pyrimidine *(**7**). Method A: A solution of **3b** (0.36 g, 10 mmol) in formamide (20 mL) was heated under reflux for 6 hours. The solid obtained was filtered off and recrystallized from benzene; Method B: Gaseous NH_3_ was bubbled through **8** (0.41 g, 10 mmol) in MeOH for 1 hour. The solid formed was collected to give **7**. White crystals (benzene); yield 75%, mp 317–319 °C; IR: 3,458, 3,380 (NH_2_), 3,174 (CH-aromatic), 2,901 (CH-aliphatic), 1,658 (C=C). MS *m/z *(%) = 389 (M+, 25.6), 249 (100), 223 (10.1), 181 (1.1). Anal. Calcd. for C_22_H_16_ClN_3_O_3_ (389.09): C, 67.78; H, 4.14; N, 10.78%. Found: C, 67.72; H, 4.10; N, 10.74%.

*Synthesis of 2-ethoxymethyleneamino-7-methoxy-4-(p-chlorophenyl)-4H-naphtho-[2,1-b]pyrane-3-carbonitrile *(**8**)*.* A mixture of **3b** (0.36 g, 10 mmol) and triethyl orthoformate (2 mL) in acetic anhydride (10 mL) was refluxed for 2 hours. After cooling, the precipitated product was filtered off and washed several times with cold EtOH. The solid obtained was filtered off and recrystallized from benzene. Colourless crystals, yield 77%, mp 211–213 °C; IR: 2,980 (CH-aromatic), 2,835 (CH-aliphatic), 2,204 (CN), 1,612 (C=N). ^1^H-NMR δ: 1.27 (t, 3H, CH_3_, *J* = 7.1 Hz), 3.78 (s, 3H, OCH_3_), 4.40 (q, 2H, CH_2_, *J* = 7.1 Hz), 5.58 (s, 1H, pyran CH), 7.24–785 (m, 9H, Ar-H), 8.67 (s, 1H, N = CH). Anal. Calcd. for C_24_H_19_ClN_2_O_3_ (418.11): C, 68.82; H, 4.57; N, 6.69%. Found: C, 68.80; H, 4.51; N, 6.62%.

### 3.3. General Procedure: Synthesis of Pyranopyrimidine Derivatives ***9*** and ***10***

A mixture of **8** (0.41 g, 10 mmol), hydrazine hydrate (5 mL, 99%) or methylamine (10 mmol) in absolute ethanol (50 mL) was stirred for 1 hour at room temperature. The solid obtained was filtered off and recrystallized from dioxane.

*10-Amino-10,11-dihydro-11-imino-3-methoxy-12-(p-chlorophenyl)-12H-naphtho[2,1-b]pyrano[2,3-d]pyrimidine* (**9**). White crystals, yield 81%, mp 256–258 °C; IR: 3,316, 3,270 (NH_2_), 3,209 (NH), 2,936 (CH-aromatic), 2,899 (CH-aliphatic), 1,647 (C=N). ^1^H-NMR δ: 3.80 (s, 3H, OCH_3_), 5.66 (s, 1H, pyran CH), 5.87 (br, 2H, NH_2_, exchangeable by D_2_O), 7.15–7.79 (m, 10H, Ar-H and NH), 8.04 (s, 1H, pyrimidine CH). Anal. Calcd. for C_22_H_17_ClN_4_O_2_ (404.10): C, 65.27; H, 4.23; N, 13.84%. Found: C, 65.25; H, 4.20; N, 13.82%.

*10,11-Dihydro-11-imino-3-methoxy-10-methyl-12-(p-chlorophenyl)-12H-naphtho[2,1-b]pyrano[2,3-d]pyrimidine* (**10**). White crystals, yield 80%, mp 255–257 °C; IR: 3,376 (NH), 3,006 (CH-aromatic), 2,980, 2,830 (CH-aliphatic), 1,620 (C=N). ^1^H-NMR δ: 3.32 (s, 3H, N-CH_3_), 3.77 (s, 3H, OCH_3_), 5.29 (s, 1H, pyran CH), 6.98–7.80 (m, 10H, Ar-H and NH), 8.01 (s, 1H, pyrimidine CH). Anal. Calcd for C_23_H_18_ClN_3_O_2_ (403.11): C, 68.40; H, 4.49; N, 10.40%. Found: C, 68.20; H, 4.41; N, 10.38%.

*Synthesis of 7-methoxy-2-(N,N-dimethylaminomethylene)-4-(p-chlorophenyl)-4H-naphtho[2,1-b]-pyrane-3-carbonitrile* (**11**)*.* A mixture of **8** (0.41 g, 10 mmol) and dimethylamine (5 mL) in ethanol was stirred for 1 hour. The white solid formed was filtered, washed with cold EtOH and recrystallized from benzene. White crystals, yield 79%, mp 218–220 °C; IR: 2,924 (CH-aliphatic), 2,190 (C≡N), 1,616 (C=N). ^1^H-NMR δ: 2.97 (s, 3H, N-CH_3_), 3.13 (s, 3H, N-CH_3_), 3.78 (s, 3H, OCH_3_), 5.40 (s, 1H, pyran CH), 7.18–7.83 (m, 9H, Ar-H), 8.42 (s, 1H, N=CH). Anal. Calcd. for C_24_H_20_ClN_3_O_2_ (417.12): C, 68.98; H, 4.82; N, 10.06%. Found: C, 68.81; H, 4.66; N, 9.98%.

### 3.4. General Procedure: Synthesis of Pyranotriazolopyrimidine Derivatives ***12–16***

A mixture of **9** (0.40 g, 10 mmol), triethyl orthoformate, formic acid, acetyl chloride or benzoyl chloride (0.01 mol) in dry benzene (20 mL), was refluxed for 3 hours. The solid obtained was filtered off and recrystallized from dioxane.

*11-Methoxy-14-(p-chlorophenyl)-14H-naphtho[2,1-b]pyrano[3,2-b][1,2,4]triazolo[1,5-c]pyrimidine* (**12**). White crystals, yield 80%, mp 260–262 °C; IR: 3,064 (CH-aromatic), 2,984, 2,844 (CH-aliphatic), 1,602 (C=C). ^1^H-NMR δ: 3.78 (s, 3H, OCH_3_), 5.42 (s, 1H, pyran CH), 7.00–7.91 (m, 9H, Ar-H), 8.63 (s, 1H, pyrimidine CH), 9.51 (s, 1H, triazolo CH). Anal. Calcd. for C_23_H_15_ClN_4_O_2_(414.09): C, 66.59; H, 3.61; N, 13.50%. Found: C, 66.50; H, 3.53; N, 13.10%.

*2-Methyl-11-methoxy-14-(p-chlorophenyl)-14H-naphtho[2,1-b]pyrano[3,2-e][1,2,4]triazolo[1,5-c]pyrimidine *(**13**). White crystals, yield 85%, mp 283–285 °C; IR: 3,074 (CH-aromatic), 2,936, (CH-aliphatic), 1,622 (C=C). ^1^H-NMR δ: 2.40 (s, 3H, CH_3_), 3.79 (s, 3H, OCH_3_), 5.43 (s, 1H, pyran CH), 6.99–7.99 (m, 9H, Ar-H), 9.51 (s, 1H, pyrimidine CH). Anal. Calcd. for C_24_H_17_ClN_4_O_2_ (428.10): C, 67.21; H, 3.96; N, 13.06%. Found: C, 67.10; H, 3.66; N, 12.99%.

*2-Phenyl-11-methoxy-14-(p-chlorophenyl)-14H-naphtho[2,1-b]pyrano[3,2-e][1,2,4]triazolo[1,5-c]pyrimidine *(**14**)*.* Pale yellow crystals, yield 70%, mp 281–283 °C; IR: 3,058 (CH-aromatic), 2,920 (CH-aliphatic), 1,626 (C=C). ^1^H-NMR δ: 3.78 (s, 3H, OCH_3_), 5.32 (s, 1H, pyran CH), 7.03–7.91 (m, 14H, Ar-H), 8.70 (s, 1H, pyrimidine CH). Anal. Calcd for C_29_H_19_ClN_4_O_2_ (490.12): C, 70.95; H, 3.87; N, 11.41%. Found: C, 70.65; H, 3.66; N, 11.27%.

*Synthesis of 11-methoxy-14-(p-chlorophenyl)-14H-naphtho[2,1-b]pyrano[3,2-e][1,2,4]triazolo[1,5-c]pyrimidine-2-ethanenitrile *(**15**). A mixture of **9** (0.40 g, 10 mmol) with ethyl cyanoacetate (10 mmol) in absolute ethanol (30 mL), was refluxed for 3 hours. The solid obtained was filtered off and recrystallized from dioxane. White crystals, yield 70%, mp 291–293 °C; IR: 2,934 (CH-aliphatic), 2,200 (C≡N), 1,604 (C=C). ^1^H-NMR δ: 3.80 (s, 3H, OCH_3_), 4.50 (s, 2H, CH_2_), 5.40 (s, 1H, pyran CH), 6.99–7.94 (m, 9H, Ar-H), 9.64 (s, 1H, pyrimidine CH). Anal. Calcd. for C_25_H_16_ClN_5_O_2_ (453.10): C, 66.16; H, 3.52; N, 15.42%. Found: C, 66.01; H, 3.41; N, 15.23%.

*Synthesis of 11-methoxy-14-(p-chlorophenyl)-2oxa-2H,3H,14H-naphtho[2,1-b]pyrano[3,2-e][1,2,4]-triazolo[1,5-c]pyrimidine* (**16**). A mixture of **9** (0.40 g, 10 mmol) with ethyl chloroformate (10 mmol) in dry benzene (30 mL) was refluxed for 1 hour. The solid obtained was filtered off and recrystallized from dioxane. White crystals, yield 76%, mp 310–312 °C: IR: 3,200 (NH), 2,988, 2,930 (CH-aliphatic), 1,638 (C=O), ^1^H-NMR δ: 3.80 (s, 3H, OCH_3_), 5.28 (s, 1H, pyran CH), 6.80–7.88 (m, 10H, Ar-H and NH), 9.64 (s, 1H, pyrimidine CH). Anal. Calcd. for C_23_H_15_ClN_4_O_3_ (431.08): C, 64.12; H, 3.50; N, 13.00%. Found: C, 63.86; H, 3.45; N, 12.81%.

*Synthesis of 10-benzalamino-10,11-dihydro-11-imino-3-methoxy-12-(p-chlorophenyl)12H-naphtho-[2,1-b]pyrano-[2,3-d]pyrimidine* (**17**). A mixture of **9** (0.40 g, 1.0 mmol), with benzaldehyde (10 mmol), piperidine (0.5 mL) and dioxane (30 mL) was refluxed for 6 hours. The precipitate was filtered off and washed several times with cold EtOH. The solid was recrystallized from dioxane. White crystals, yield 71%, mp 255–257 °C; IR: 3,261 (NH), 2,988, 2,930 (CH-aliphatic), 1,652 (C=N), ^1^H-NMR δ: 3.79 (s, 3H, OCH_3_), 5.66 (s, 1H, pyran CH), 6.80–7.88 (m, 15H, Ar-H and NH), 8.27 (s, 1H, N=CH), 9.64 (s, 1H, pyrimidine CH). Anal. Calcd. for C_29_H_21_ClN_4_O_2_(492.14): C, 70.66; H, 4.29; N, 11.37%. Found: C, 70.54; H, 4.04; N, 11.21%.

### 3.5. Antimicrobial Assay

Inoculums of the bacterial and fungal culture were prepared. To a series of tubes containing 1 mL each of naphthopyran compound solution with different concentrations and 0.2 mL of the inoculums was added. A further 3.8 mL of sterile water was added to each of the test tubes. These test tubes were incubated for 24 hours at 37 °C and observed for the presence of turbidity. This method was repeated by changing naphthopyran compounds for the standard drugs ampicillin and ketoconazole for comparison.

## 4. Conclusions

Our interest in the synthesis of such compounds was to focus on their study as antimicrobial agents as a part of our program which aimed at the development of new heterocyclic compounds as more potent antimicrobial agents. In this paper we revealed the synthesis of some new naphthopyran, naphthopyranopyrimidine and naphthopyranotriazolopyrimidine derivatives and the antimicrobial evaluation of all the novel compounds. The structures of these compounds were elucidated on the basis of IR, ^1^H-NMR, ^13^C-NMR and MS data. Evaluation of the new compounds established that **3a–e**, **12**–**14** and **16** showed improved antimicrobial activity.
